# In-situ data of occupant behavior and thermal comfort in 11 offices in a naturally ventilated building during 6 summers

**DOI:** 10.1016/j.dib.2025.111722

**Published:** 2025-05-29

**Authors:** Simon Bal-Fontaine, Pierre Bernaud, Kevin Campagna, Hugo Coulandreau, Aurélie Foucquier, Arnaud Jay, Merveil Muanda Lutete

**Affiliations:** a Université Grenoble Alpes, CEA LITEN, INES, Le Bourget du Lac, France; b Université Savoie Mont-Blanc, CNRS, LOCIE, 73000 Chambéry, France; c Centre Scientifique et Technique du Bâtiment (CSTB), 24 Rue Joseph Fourier, 38400 Saint Martin-d'Hères, France

**Keywords:** Openings, Doors, Window operation, Fan usage, Summer comfort, Passive cooling, Monitoring, Experimental data

## Abstract

As summer heatwaves become more intense and more frequent, achieving thermal comfort in buildings without relying on air conditioning has become critical to meet CO₂ emission targets. To address this, a field study was conducted in an office building at INES, Le Bourget-du-Lac, France. The bioclimatic design of the building takes advantage of the local climate, influenced by mountain ranges and Bourget Lake, to minimize overheating while ensuring occupant comfort thanks to solar control, natural ventilation and thermal inertia. It optimizes natural ventilation through features such as a green atrium with automated openings and internal leafs. Pedestal fans completed this strategy to maximize occupant comfort without air-conditioning.

Data were collected over six years (2017–2022) through sensors networks and thermal comfort surveys thanks to a Human Machine Interface (HMI), which has been developed and improved over the different campaigns. The surveys gather occupant perception of comfort, clothing details, and the effect of draughts, while sensors monitored door and window openings, office temperatures and fan use. Instrumentation includes temperature sensors connected to computers, plug-in power sensors for fans, and openings status sensors. Data were recorded every 5–10 min using a Jeedom home automation system linked to a Raspberry Pi, with a Python script transferring data to a central database. Across the study, up to 29 participants contributed annually, generating datasets ranging per year from 250 to 17,500 responses.

This dataset supports research into the relationship between thermal comfort, occupant perception, and passive cooling strategies, such as natural and mixed-mode ventilation. It provides insights into variations in occupant comfort and behavior across similar offices environment and forms a basis for studying building overheating, operational performance, and the effectiveness of passive cooling strategies. This dataset also facilitates the development of ventilation control algorithms and cascade cooling strategies by analyzing the conditions under which occupants use desk fans, helping optimize mixed-mode ventilation systems for comfort and efficiency.

Specifications Table

**Table:** 

Subject	Energy: Renewable Energy, Sustainability and the Environment
Specific subject area	In-situ data of occupant behavior and thermal comfort in offices in a naturally ventilated building in France*.*
Type of data	Table Raw.
Data collection	Data were collected over six years (2017–2022) through thermal comfort surveys and sensors networks. The surveys gather occupant perception of comfort, clothing details, and the perception of draughts, while sensors monitored windows and doors openings, offices temperatures, and fans usage. Monitoring includes temperature sensors connected to computers, plug-in power sensors for fans, and opening status sensors. Data were recorded every 5–10 min using a Jeedom home automation system linked to a Raspberry Pi, with a Python script transferring data to a central database*.*
Data source location	CEA offices @ Helios Building, INES 60 avenue du Lac Léman 73360 Le Bourget-du-Lac France (45.640804; 5.875095)
Data accessibility	Repository name: National french repositiory for research “Recherche Data Gouv” Data identification number: DOI: 10.57745/MWV5H4 Direct URL to data: 10.57745/MWV5H4 Instructions for accessing these data: … See the direct URL above
Related research article	None

## 1 Value of the Data

• This dataset enable research into a deeper understanding of the relationship between comfort, perception, and actions related to various passive cooling methods (natural and mixed-mode ventilation). It provides insights into the potential benefits of desk fan usage on perceived thermal comfort, considering their power and airspeed.
• The data address the need to complete the ASHRAE Comfort Database [1] and the Occupant Behavior Database [2]. It supports the requirement for experimental data on comfort in natural and mixed-mode ventilation systems. As highlighted in IAE 69 [3] and Annex IAE 79 [4], it also underscores the need for more detailed datasets on occupant behavior, particularly concerning comfort and occupant perception. In addition, these data also meet the needs of these two annexes for data on occupant behavior in summer conditions and in countries such as France, which are not currently available in existing datasets.
• Enriching existing datasets is crucial for evaluating, validating, and developing both existing and new models of comfort and occupant behavior during summer [4]. Incorporating factors such as occupant comfort and clothing into behavior models could significantly advance behavior modelling in building design and operations, potentially reducing performance gaps and construct comfortable naturally ventilated buildings [5].
• The data allow researchers to investigate the diversity levels of occupant behaviors and comfort in different offices exposed to similar conditions and work environments [6]. By analysing intra- and inter-office comfort perceptions, it can reveal variations in how occupants experience and respond to their environment.
• These data provide a foundation for studying building overheating and operational performance in naturally ventilated office buildings during the summer. It offers a basis for analysing temperature data in relation to building characteristics and occupant behavior, which can be used to evaluate the effectiveness of passive cooling designs.
• Researchers can utilize this data to develop and refine natural ventilation control algorithms, potentially improving the performance of mixed-mode ventilation systems and aligning them with occupant comfort preferences. Indeed,these data can permit to study at which temperature, occupants use desk fan to improve their comfort. That can help researchers to develop cascade-cooling strategy [7].

## 2 Background

Longer and more intense summer heatwaves will make summer comfort one of the main building issues. As a result, the overheating periods in buildings will increase sharply and the main concern for occupants’ comfort will be in summer. To have comfortable buildings in summer and meet national targets for CO2 emissions [8], the answer is not always to install air conditioning in buildings but to better design naturally ventilated buildings. For that, it is necessary to evaluate, model and understand thermal comfort and the occupant behavior with real data. Unfortunately, research on this subject is limited by the fact that there is very little to be found in the literature and databases on this type of building [2].

That is why, a field study has been conducted in the Helios building @INES, designed to optimize natural ventilation, to collect thermal comfort perception, indoor and outdoor conditions and occupant interactions with fans, windows, and doors. The dataset aims to be shared to scientific community to develop, test and model thermal comfort and occupant behavior models.

Based on this dataset a first article has been published [9], related to the development of an innovative approach for predicting indoor thermal comfort in an office building.

## 3 Data Description

### 3.1 Dataset structure

Data have been structured to follow the template of the ASHRAE Occupant Behavior Dataset [2], so that this database can be accessed through their API in the future. Thus, data were break down into five files. The three first files (*Window_Status, Fan_Status*, and *Door_Status*) aim to describe the status of windows, fans, and doors of the monitored offices. These data come from sensors installed in the offices. The fourth file *Outdoor_Measurement* contains all available meteorological data during the measurement periods, collected from a weather station installed near the offices. Finally, the file *EvalConfort* contains the responses from the survey given to the occupants of the monitored offices of all six years.

### 3.2 Dataset variables

**Fig. 1:**
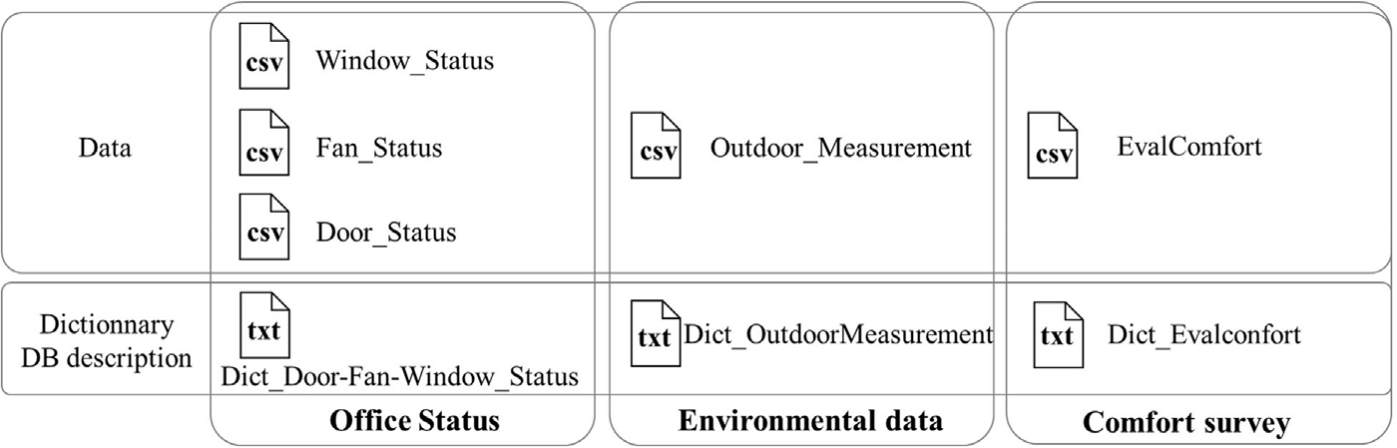
Dataset structure and files composing the dataset.

The variables contained in each file are detailed in Table 1 for the *Window_Status, Fan_Status*, and *Door_Status* files, Table 2 for the *Outdoor_Measurement* file, and Table 3 for the *EvalConfort* file, and are listed in text files as Python Dictionary in Dict_Door-Fan-Window_Status, Dict_OutdoorMeasurement and Dict_Evalconfort as highlighted in Fig. 1. All the openings that are in the buildings are explained in the part “Experimental in Design, Materials and Methods” and are summarized in the Table 4.

**Table 1:** Variables composing window_status, fan_status, and door_Status files.

	Value	Description
Status_ID	OUV_FEN: window OUV_VFE: external office leaf OUV_POR: door OUV_VPO: internal office leaf PUI_VEN: fan	Type of window, door or fan
Date_Time	Format: dd/mm/yyyy HH:MM:SS	UTC date and time of the year
Status	0: Close for a window or a door 1: Open for a window or a door Float for fan	Status of the window or door Electric power for fan
ID	String	Sensor name
Room_ID	Integer	ID corresponding to the room (see map)
Building_ID	0	Only one building is considered in this dataset

**Table 2:** Variables composing Outdoor_Measurement file.

	Value	Description	Number of non null point
Outdoor_Measurement_ID	‘Weather_Station_01’	Only one weather station is considered in this dataset	315,504
Date_Time	Format: dd/mm/yyyy HH:MM:SS	UTC date and time of the year	315,504
Outdoor_Temp[C]	Float	Dry bulb outdoor air temperature in [°C]	312,950
Outdoor_RH[%]	Float	Outdoor Relative Humidity in [%]	312,967
Wind_Speed[m/s]	Float	Wind speed in [m/s]	294,470
Wind_Direction[deg]	Float	Wind direction in [deg]	312,796
Outdoor_Air_Pressure[hPa]	Float	Outdoor Air Pressure in [hPa]	312,967
Solar_Radiation[w/m2]	Float	Global horizontal solar radiation in [W/m²]	312,964
Building_ID	Only ‘0’	Only one building is considered in this dataset	315,504

**Table 3:** Variables composing EvalConfort file.

	Values : English translation (French Original version)	Description	IHM_ version	Number of non null point
Date_Time	Format: dd/mm/yyyy HH:MM:SS	Date and time of the year	[[1], [2], [3]]	295,508
Tint_degC	Float	Indoor air temperature measured thanks to sensor plugged to each volunteer’s laptop	[[1], [2], [3]]	293,679
Thermal_Comfort	Float between -3: very cold (très froid) -2: cold (froid) -1: a bit cold (un peu froid) 0: comfortable (confortable) 1: a bit warm (un peu chaud) 2: warm (chaud) 3: very warm (très chaud) 10: unchanged (inchangé)	Occupant's answer to the thermal comfort question	[[1], [2], [3]]	295,508
clothing	0: sweater & pants (Pull & pantalon) 1: long sleeves & pants (Manches longues & pantalon) 2: short sleeves & pants (Manches courtes & pantalon) 3: short sleeves & shorts/dress (Manches courtes & short ou jupe) 10: unchanged (inchangé)	Occupant's answer regarding clothing choice based on temperature regulation needs	[1]	121,93
FanState	0: none () 1: low () 2: high () 10: unchanged ()	Occupant's answer for fan airflow settings	[1]	121,93
WindowState	0: closed (Fermée) 1: open (ouverte) 10: unchanged (inchangée)	Occupant's answer for window open/close	[1]	121,93
BlindState	0: no 1: yes 10: unchanged	Occupant's answer regarding solar shading (blinds)	[1]	121,93
Occupancy	0: less than an hour 1: more than an hour 10: unchanged	Occupant's answer for presence in their office	[1]	121,93
Room_ID	Integer	ID corresponsponding to the room	[[1], [2], [3]]	287,891
Personal_ID	Integer	ID corresponding to the volunteer filling the HMI	[[1], [2], [3]]	292,550
Gender	F: Female M: Male ‘nan’: Undefined / unknown	Gender of the volunteer	[[1], [2], [3]]	294,664
Clo_calculation	Float	Clo variable calculate according to norm EN ISO 9920	[3]	477,36
Air_Flow	Float	response to occupant's sensation of draught	[2,3]	283,315
Upper_clothing_layer_1	0: No sleeves (sans manches) 1: Short sleeves (manches courtes) 2: Long sleeves (manches longues) 10: unchanged	Occupant’s answer for upper clothing layer (first layer)	[2,3]	283,315
Upper_clothing_layer_2	0: None (Aucune) 1: Long sleeves (manches longues) 2: Sweater (pull) 10: 'unchanged	Occupant’s answer for upper clothing layer (second layer)	[2,3]	283,315
Lower_clothing	before 2020: {0: Shorts, 1: Dress/Skirt, 2: Pants, 10: unchanged}, {0: Short, 1: Robe/Jupe, 2: Pantalon, 10: inchangé} 2020 and after: {0: Shorts, 1: Longer Shorts, 2: Light pants, 3: Pants, 10: unchanged} {0: Short, 1: Mi-court, 2: Pantalon léger, 3: pantalon, 10: inchangé}	Occupant’s answer for lower clothing (pants, shorts, or dress)	[2,3]	283,315
Footwear	before 2020: {0: Shoes, 1: Sandals/Flip-flops, 10: unchanged} {0: Chaussures, 1: Sandalles/Nus-pieds, 10: inchangé} 2020 and after: {0: Sandals/Flip-flops, 1: Shoes without socks, 2: Shoes with socks, 3: Shoes with thick socks, 10: unchanged} 0: Sandales/ Nus-pieds, 1: Chaussures sans chaussettes, 2: Chaussures avec chaussettes, 3: Chaussures avec chaussettes épaisses, 10: unchanged}	Occupant’s answer for footwear (shoes or sandals)	[2,3]	283,315
Face	0: No Face mask (Sans masque) 1: Face mask (Avec masque) 10: unchanged	Occupant’s answer for face mask usage status	[3]	47,736
Tclim_degC	Float	Air conditioning outlet temperature of office 3910	[3]	1,709

**Table 4:** Solar protections and natural ventilation openings recap, with their type of control and their legend.

Object	Legend	Type of control
Internal office leaf	1	Manual
External office leaf	2	Manual
External atrium louvers	3	Automatic
atrium glass roof	4	Automatic
Internal office blind	5	Manual
Adjustable office solar shadings	6	Manual
Internal atrium louvers	7	Automatic
Internal atrium blinds	8	Automatic
External adjustable solar shadings	9	Automatic

Window, fan, door status data available: Office Status

Data of offices status including windows, doors, and fans, manually controlled by occupants, were collected with sensors during 2018, 2019 and 2020. There are no status data for the year 2017, as the sensors were not yet installed, but in the *EvalConfort* file for the 2017 year, those information were collected at the same time of the survey. The reliability of the information collected through the occupant feedback is less robust than with status sensors as some human errors sometimes occurred when filling this survey. There are also no data for the years 2021 and 2022, as the data of the sensors were not maintained during those years. Window_Status, Fan_Status, and Door_Status files have respectively, 287,896 points, 815,011 points (ranging from 0 to 117W) and 278,840 points.Survey Data available: EvalConfort

**Fig. 2:**
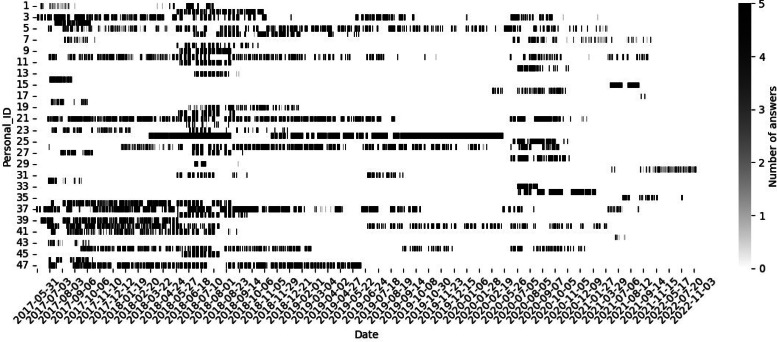
Number of answers per day for each individual of the dataset (data from EvalComfort).

Fig. 2 represents the responses to the questions according to the time of year. 47 different individuals responded to the survey over the course of the 5 years of the study (from 2017-06-13 to 2022-11-14). However, the responses were uneven: across the years (some individuals participating occasionally or being involved for only a year due to short-term contract), across the months (due to vacations), and across the days (due to working period outside the office or lack of motivation).

**Fig. 3:**
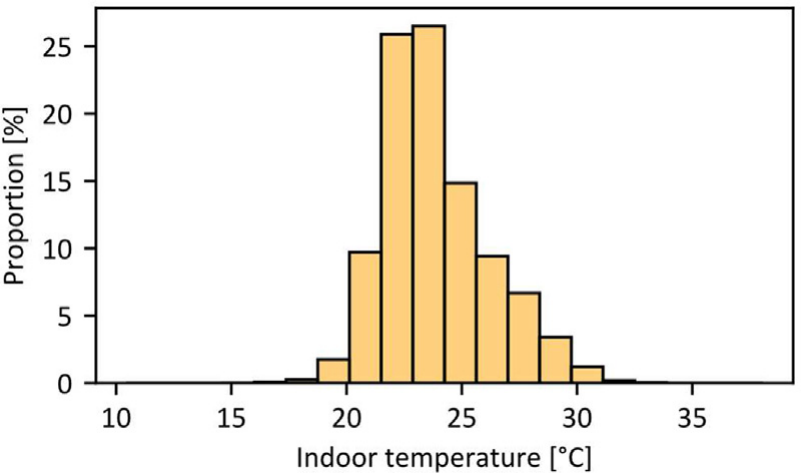
Distribution of indoor temperature in all offices monitored.

**Fig. 4:**
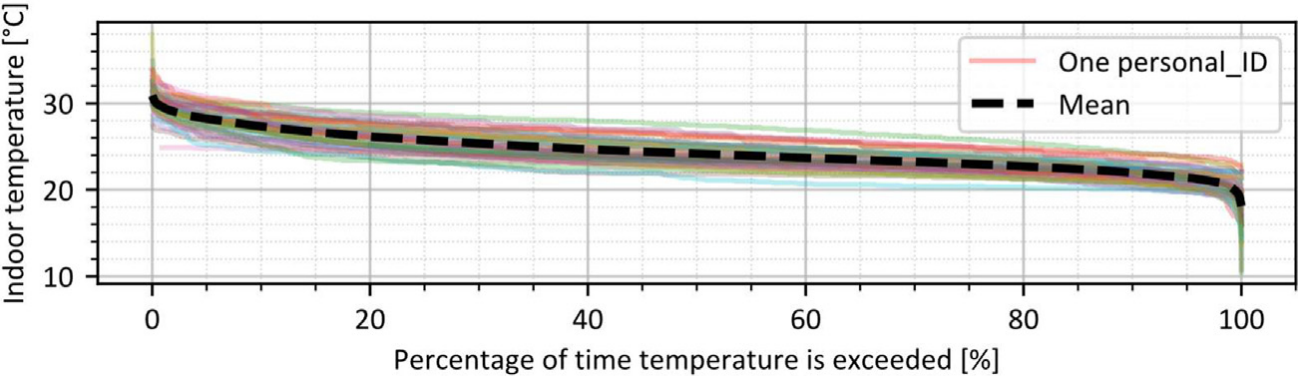
Duration curves per Personal_ID measured during the monitoring campaign.

To better understand the database and the range of indoor environmental conditions, the distribution of indoor temperature measurements throughout the entire monitoring campaign is presented in Fig. 3, and the indoor temperature duration curve is shown in Fig. 4. Most indoor temperature values fall between 18 °C and 32 °C. As all indoor temperature data are provided in their raw form, values that fall outside a plausible range have been intentionally retained in the dataset. This allows future researchers to apply their own criteria for data cleaning and filtering based on their specific needs and assumptions. As an example, only 995 temperature values fall outside the 5 °C to 40 °C range out of a total of 293,679 measurements, representing approximately 0.3 % of the dataset.

**Fig. 5:**
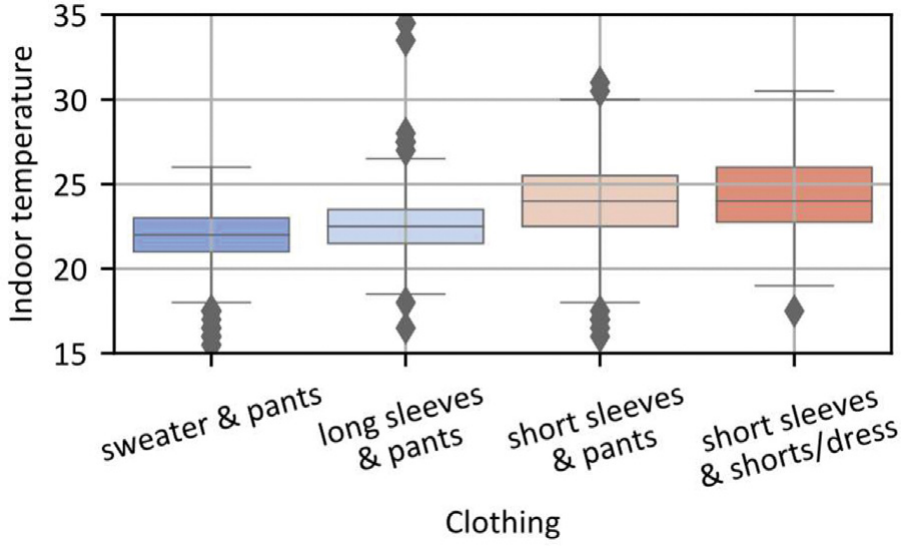
Boxplot of the clothing level ('clothing' variable) of all occupants in function of the indoor temperature at the time of the answer.

**Fig. 6:**
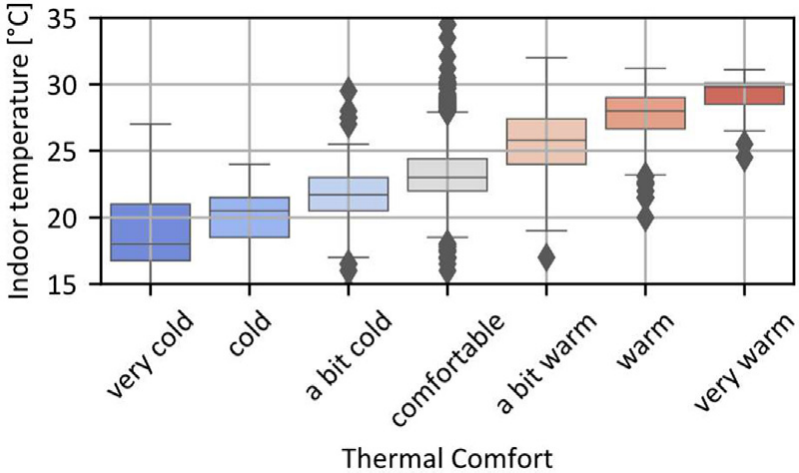
Boxplot of the thermal comfort ('Thermal_Comfort' variable) of all occupants in function of the indoor temperature measure at the time of the answer.

To illustrate the type of information that can be extracted from the dataset, Figs. 5 and 6 respectively display boxplots of all occupants' responses regarding their clothing level (‘clothing’ variable) and their thermal comfort sensation (‘Thermal_comfort’ variable), as a function of the indoor temperature at the time of their response.

### 3.3 Specificity of office 3102

It should be noted that Tclim_degC variable corresponds to the output temperature of the air conditioning system in the only air-conditioned office in the building, number 3102. This value was recorded each time occupant with Personal ID 33 responded to the survey. Consequently, this value is available in the Tclim_degC column of the *EvalConfort* file, in the response’s lines of occupant ID 33. The full range of temperature data extends from 0 °C to 30 °C, with most values (99 %) falling between 20 °C and 30 °C

### 3.4 Weather description (Outdoor measurement)

**Fig. 7:**
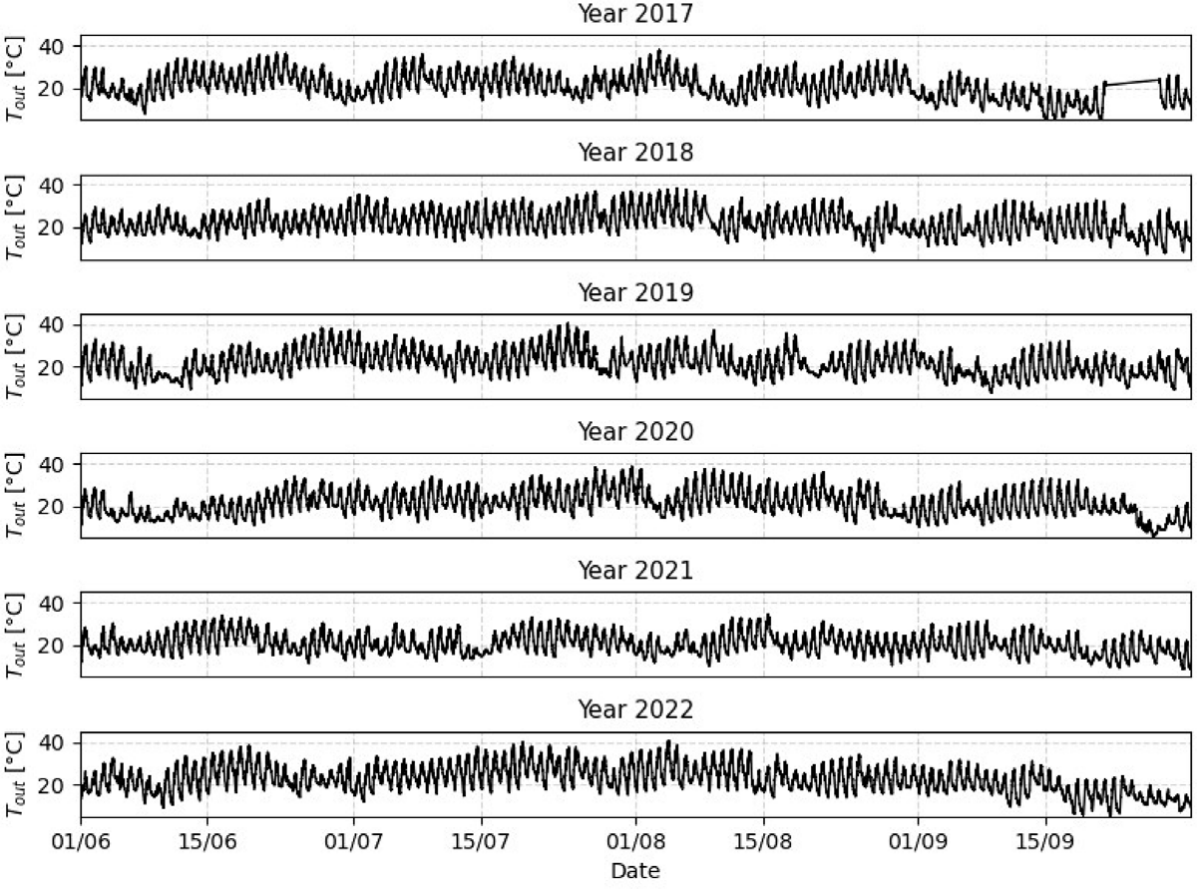
Outdoor Temperature for hot season.

Weather conditions were measured using a weather station located on the experimental site, a few hundred meters from the building. To ensure comprehensiveness and to maximize the use of available data, six years data with a 10 minute time step are included in the dataset, even though other datasets primarily focus on the summer season, some of the volunteers give their feedback all the yearlong, so the weather data are continuous. Only four days of data are missing, from the 13^th^ to the 16^th^ of February 2021 due to a failure in the acquisition system. Some other data are missing for certain periods of time; for instance, no outdoor temperature was recorded for one week in mid-September 2017. To describe the climate, the outdoor temperature is plotted in Fig. 7 for the hot season of each year (from the beginning of June to the end of September).

Moreover, the weather station does not measure IAQ, Particle_Level[ug/m3], Particle_Type, or Precipitation. These columns contain no associated values in the dataset but are included to comply with the template of the Global Occupant Behavior Database.

For details about weather station sensors, refer to Table 5.

### 3.5 Experimental design, materials and methods

#### 3.5.1 Building presentation

**Fig. 8:**
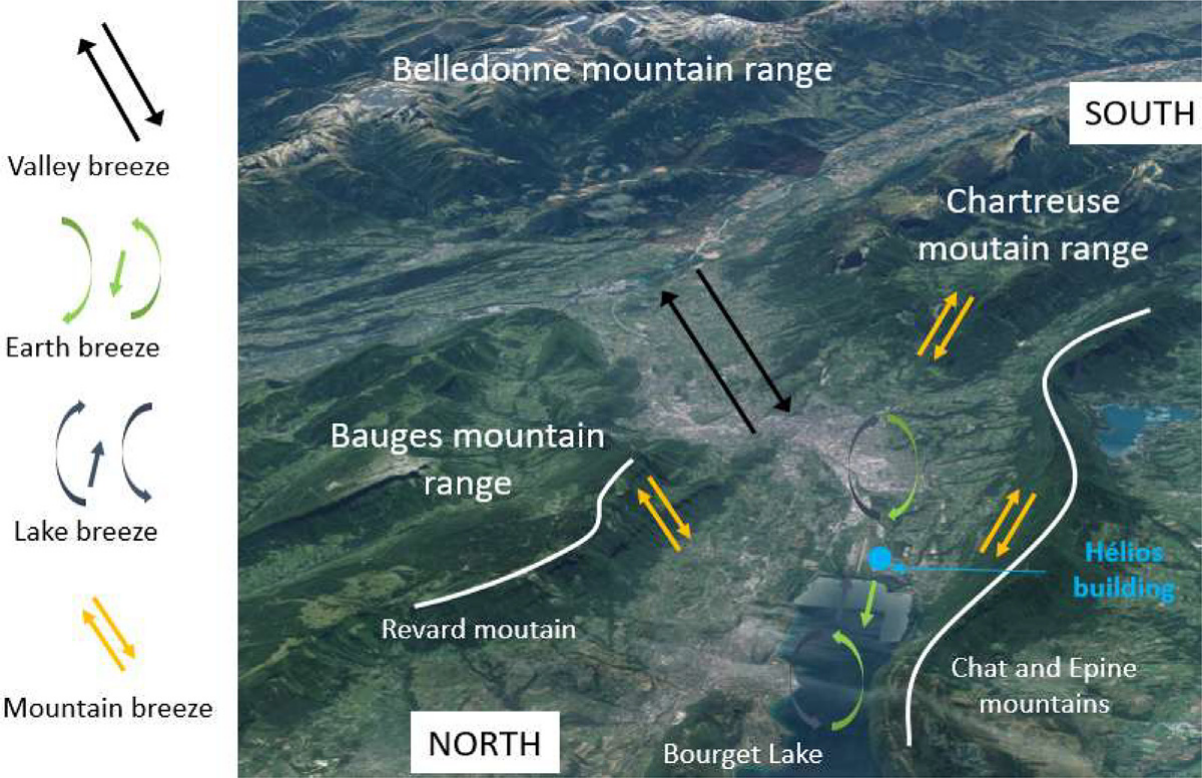
Helios building micro-climate.

The data were collected in the Helios building (45.640804; 5.875095), located in Le Bourget-du-Lac, France, at the National Institute of Solar Energy (INES), in the French Alps. According to the Köppen climate classification, the site experiences a “CfB” climate, characterized by temperate conditions with no distinct dry season and mild summers. The local climate is significantly influenced by the surrounding mountain ranges and Bourget Lake (see Fig. 8). Predominant winds follow a north-south axis, with a diurnal valley breeze directed southward and a nocturnal lake breeze moving northward.

**Fig. 9:**
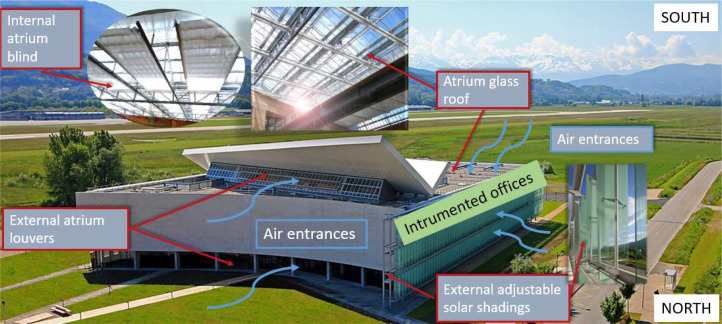
Exterior of the Helios building.

**Fig. 10:**
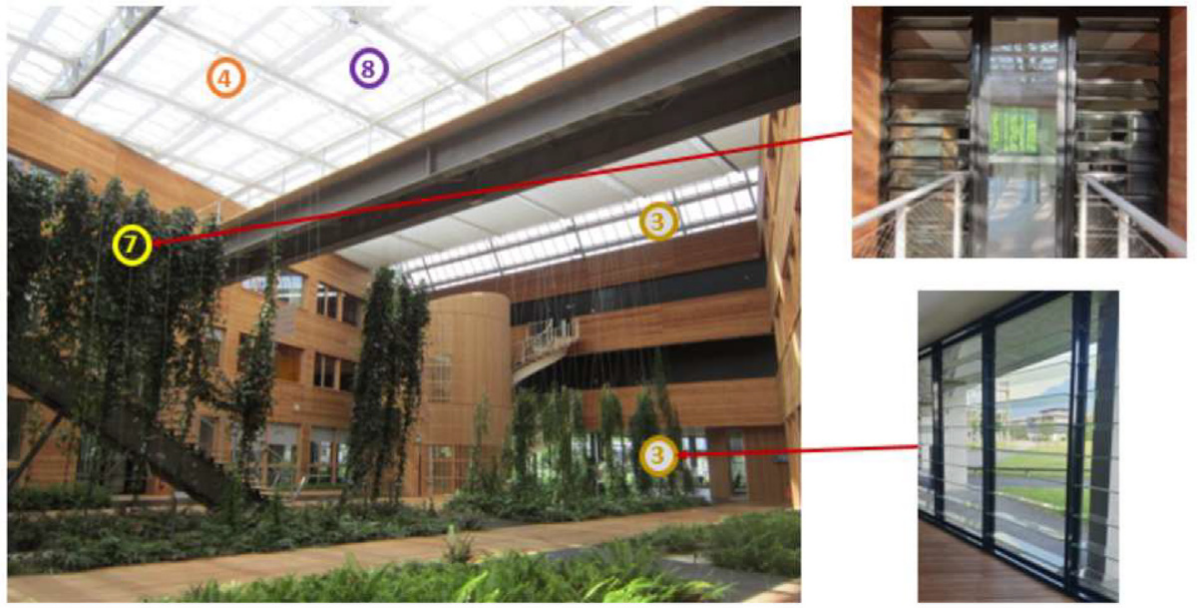
Green atrium of Helios building with (3) external atrium louvers, (4) opening atrium glass roof, (7) internal atrium louvers, (8) internal atrium blinds.

The Helios building has been designed in a bioclimatic way, taking advantage of the local climate to optimize natural ventilation potential and avoid the use of air conditioning. It has a green atrium which, thanks to its automatic openings on the roof and the north façade, acts as a buffer space to take advantage of the fresh air brought by the nocturnal lake breeze (see Fig. 9). The atrium also has internal louvers (see Fig. 10) to create draughts in the office corridors and then through the offices when the atrium temperature is lower than the office temperature. Finally, the atrium has automatic internal blinds on its roof and there are automatic solar shading devices along the entire west facade to minimize solar gain in the building.

**Fig. 11:**
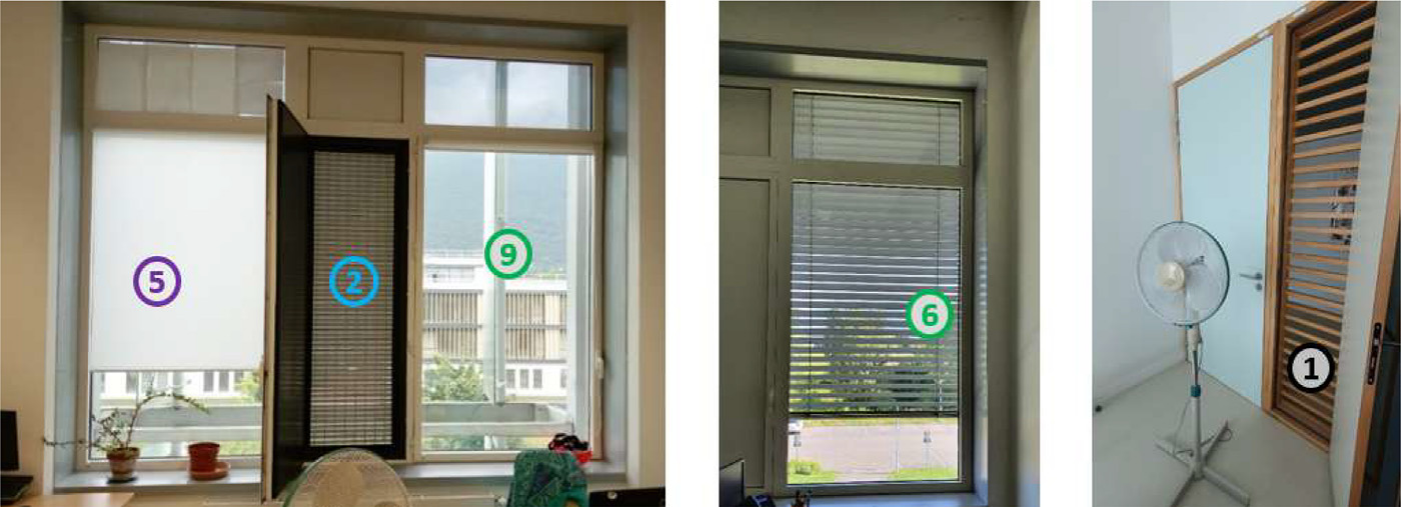
West oriented office (on the left) and South oriented office (on the center) in Helios building with (5) internal office blind, (2) external office leaf, (6) adjustable office solar shading, (1) internal office leaf, (9) external adjustable solar shading.

**Fig. 12:**
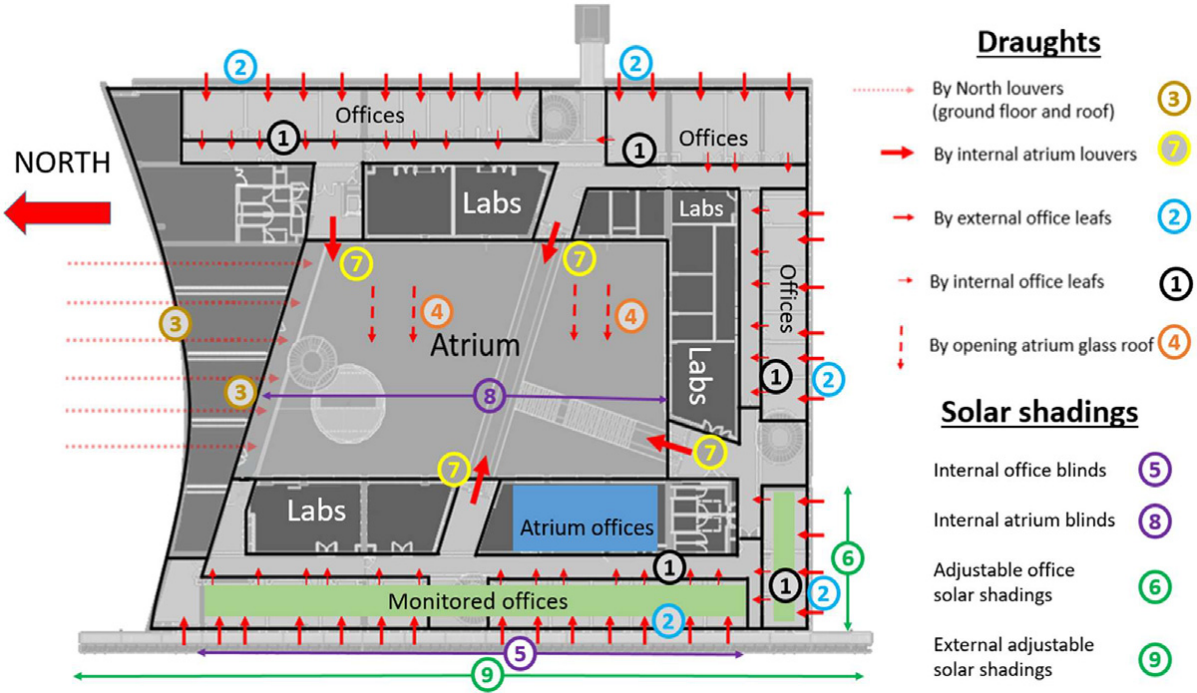
Plan of the third level Helios building with draughts ways and the related openings, and different types of solar shadings.

The offices are also designed to maximize natural ventilation, with internal and external louvers that avoid security issues while allowing draughts to pass through at night. Monitored offices are located on the West wing of the building. Windows overlooked for most of them the exterior West facade, except one which overlooked the South exterior façade and one for which window overlooked the Atrium. Louvers on the West facade have internal blinds (in addition to the external adjustable solar shadings) and those on the South façade have manually controlled adjustable sunshades (see Figs. 11 and 12). Pedestal fans are also installed in each office. They have 3 different speeds and an oscillating or fixed mode.

Fig. 12 shows the different draughts possible in the building, the different solar shadings installed, and where all the atrium and office openings are located. We can see the third level of the building, where the offices we are interested in are located, i.e. the monitored offices on the west and south facades of the building and the atrium offices, which only overlook the atrium. We can also see the difference of solar shadings between South offices and West offices

Table 4 summarizes all the openings and solar protections in the Helios buildings, their control type and their associated legend, in order to better understand how natural ventilation works in this building.

The offices are occupied by researchers working in the field of building energy. The building is open from 7 a.m. to 8 p.m., with most workers arriving between 8 a.m. and 9 a.m. and leaving between 5 p.m. and 6 p.m.

#### 3.5.2 Thermal comfort survey

The dataset comes from thermal comfort campaigns carried out during 6 summers (2017 to 2022) in the building described previously. Even if the campaign was design to collect data during summer, it was run for the whole year for some people. During these six years, a Human Machine Interface (HMI) has been developed and installed on volunteers’ office computers to capture the thermal comfort sensations of office workers. A pop-up opens on an hourly basis on the computer desktop to ensure regular participation of the volunteers.

The number of participants varies from year to year as it's based on people volunteering. So, there was, per year, a maximum of 29 people and a minimum of 4 people, giving for one year a maximum of about 17,500 answers and a minimum of about 250 answers. Here we consider that volunteers are typing, so the metabolic rate is 1,1 MET, which corresponds to light sedentary activity as defined in ASHRAE 55 and ISO 8996.

#### 3.5.3 First version

**Fig. 13:**
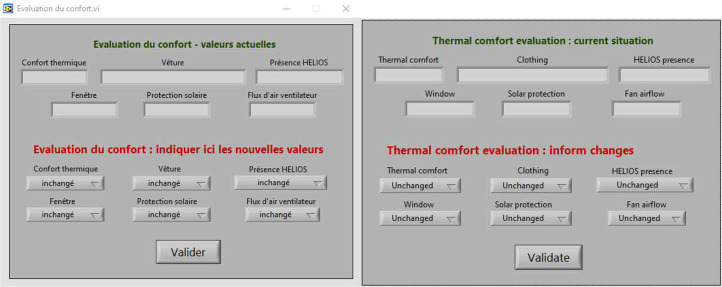
First Human-Machine Interface (HMI) used to collect data from summer 2017 to spring 2018 (left original version in French (used to collect data). Right, translated version in English for the purpose of this article).

The first version of the HMI (see Fig. 13) was used from 2017 to the spring 2018. In this version, users have to indicate their thermal comfort on a scale of seven values, from very cold to very hot. For clothing, they have to choose from four options representing the main types of clothing, i.e. jumper and trousers, long-sleeved and trousers, short-sleeved and trousers, and short-sleeved and shorts or dress. They have to answer whether they have been in their office for more or less than an hour, whether the windows and blinds are closed or open, and whether there is an air flow (important, low or none) due to the fan installed in the office.

#### 3.5.4 Second version

**Fig. 14:**
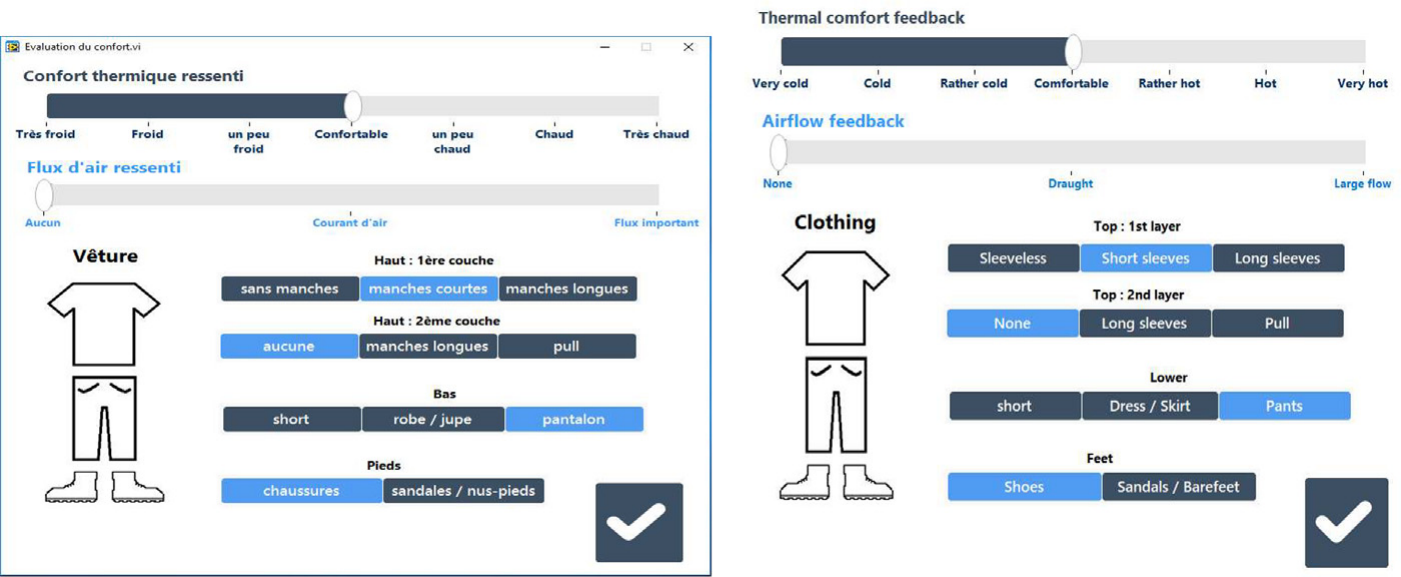
Second Human-Machine Interface (HMI) used to collect data from summer 2018 to summer 2019 (left original version in French (used to collect data). Right, translated version in English for the purpose of this article).

The second version of the HMI (see Fig. 14) was then used from summer 2018 to summer 2019. Some of the information that users need to respond to is now provided by sensors installed in the offices to make it easier to enter information into the HMI. This installation is described in the next section, “Office monitoring”.

In this version of the HMI, users can select their different garments separately to get a more accurate clothing value. The airflow is now also related to both the fan and the window opening, and users have to answer whether the amount of draught is important, low or zero. This allows us to see if the fan or window opening have an impact on thermal comfort.

#### 3.5.5 Third version

**Fig. 15:**
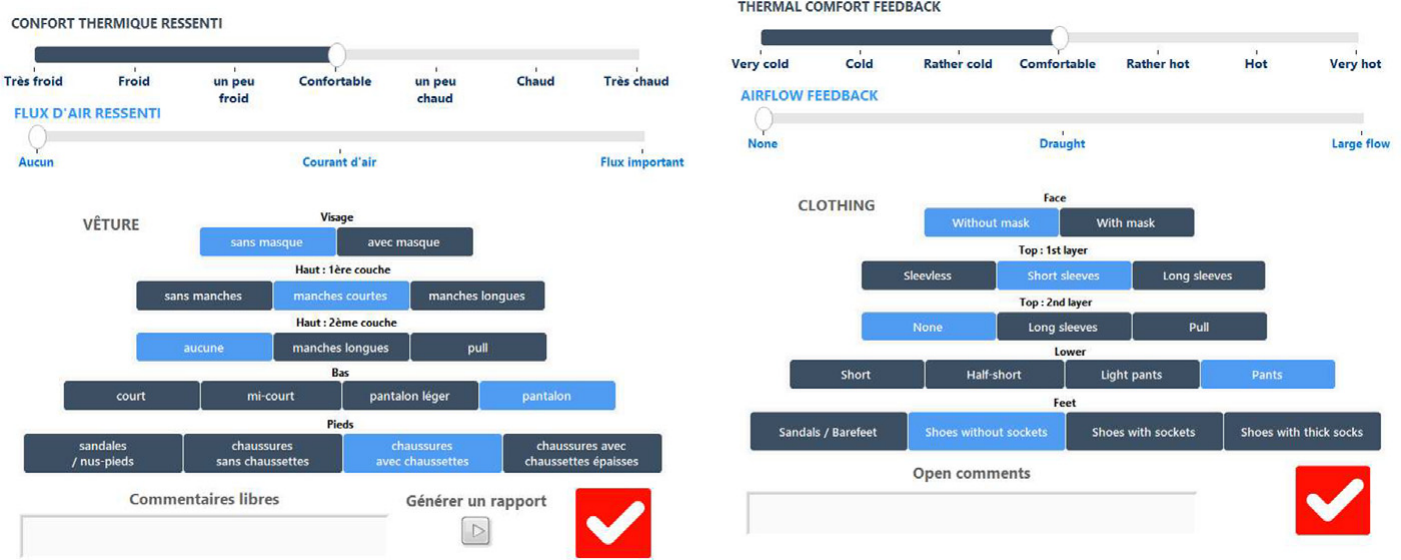
Third Human-Machine Interface (HMI) used to collect data from 2020 (left original version in French (used to collect data). Right, translated version in English for the purpose of this article).

For the next three summers (2020, 2021 and 2022), a third version of the HMI has been developed and deployed on volunteers laptop (see Fig. 15). The main update is the calculation of the clothing which now follows the NF EN ISO 9920 standard [10] in order to better understand the impact of this parameter on the thermal comfort of the occupant (Table 3).

#### 3.5.6 Office monitoring

**Fig. 16:**
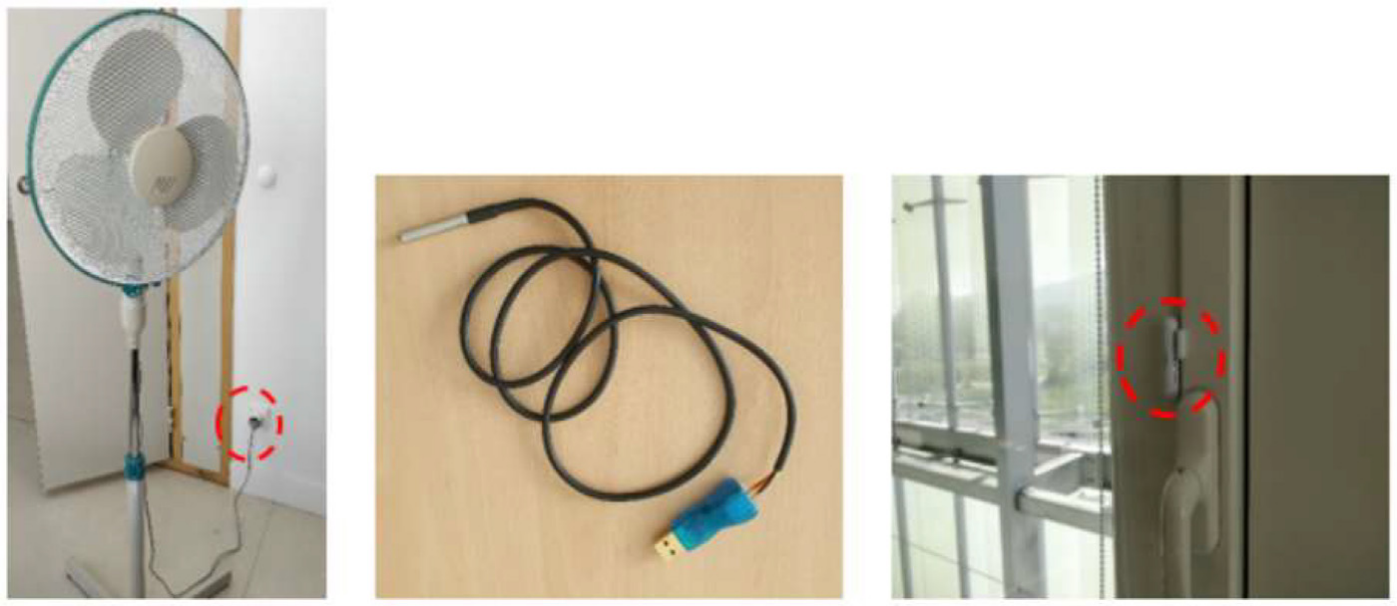
Sensors installed in the office: power meter plugs (left), temperature sensor plugged to volunteers laptop (middle), opening sensors (right).

This dataset aims to better understand thermal comfort and related occupant behavior in naturally ventilated buildings. The indoor temperature sensors installed in offices has been installed under the desk, at a height of approximately 50 cm, to avoid solar irradiation and to keep them away from computers and other electrical equipment. Each sensor is connected to the volunteer dock station. Each volunteer connects its laptop to this dock session when arriving at his/her desk. From summer 2018, connected plugs are used to have the power data of pedestal fans (shown on the left in Fig. 16). This allows us to determine when the fans are being used and at what speed, to deduce the airflow speed. Please note that the pedestal fans are RS Pro Heavy Duty Floor Fans with three 400 mm blades and three speed settings. If you want to calculate an approximate air speed, you will find the associated power in the data. Offices in the building are monitored with opening status sensors on all windows, doors or leafs (see right picture of Fig. 16). These status sensors are set up in the monitored offices of the West part presented in Fig. 12 and in office number 3092 on the south façade of the same floor. In the atrium offices (see Fig. 12), the thermal comfort HMI is also used, but only the office temperature sensor is added. In fact, these offices have an active cooling system and only one window per office facing the atrium. The active cooling, installed in offices facing the atrium, is almost never used by the occupants, as the office temperature remains quite low thanks to the atrium. However, we put a temperature sensor on the computer to measure the office temperature and another on the active cooling outlet to see if it is being used or not.

Table 5 shows the types and models of sensors, their operating range, resolution and data unit, and the number of sensors installed throughout the building. It should be noted that all the windows are single casement window with enough space to open them entirely. Concerning window and door leafs, they are fixed as we can see on Fig. 11. For external louvre, their opening rate are almost 100 % as we can see on Fig. 10.

**Table 5:** Information of office sensors.

Parameter	Sensor model	Range	Resolution	Unit	Number of sensors
Office température	DS18B20	−55 °C to 125 °C	± 0,1 °C	°C	29
Opening status	FIBARO Door/Window sensor 2	None	ON/OFF	ON/OFF	56
Fan power	FIBARO Wall Plug Type E	Up to 2,5 kW	± 0,1W	W	11

The data collection is managed by a Jeedom home automation server hosted on a Raspberry Pi. A USB Z-Wave receiver is plugged into The Raspberry Pi to communicate with Z-wave sensors. Power meter plugs are used as repeaters to transmit opening status data to the Jeedom server using the Z-wave radio protocol. Data collection is made every 5 minutes for the opening status and fan sensors and every 10 min for the office temperature sensors. In addition, indoor temperature measurements were also recorded at the exact moment of each occupant vote submission. Finally, a Python script transfers the data to a centralize online database to facilitate its use.

#### 3.5.7 Weather conditions measurements

**Fig. 17:**
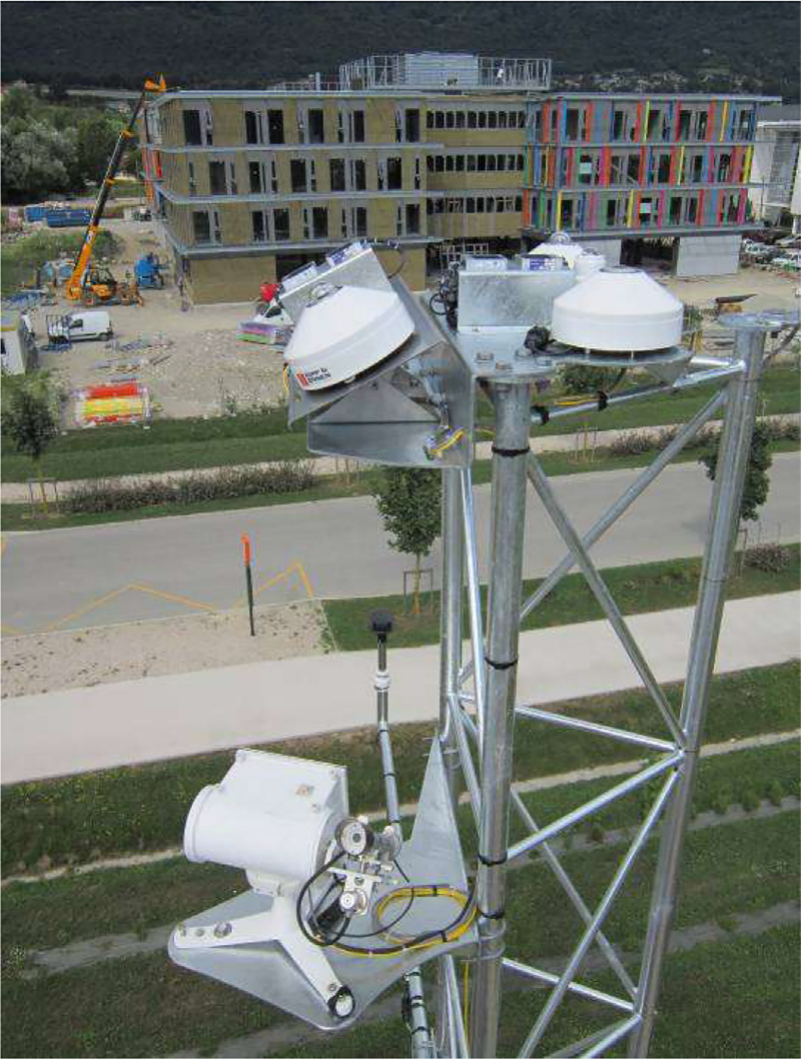
Sensors installed on weather station.

The weather conditions measurements come from a weather station (see Fig. 17) close to the HELIOS building (45,641547°; 5,874636°), at 12 m high and far from any buildings. The pyranometer is at the maximum height (12 m high), the anemometer is at 10 m, and the air temperature and humidity sensors are at 1,5 m. Table 6 list the sensors installed sensors whose values can be found in the data file *Outdoor_Measurement*.

**Table 6:** Information of weather station sensors.

Parameter	Sensor model	Range	Resolution	Unit
Dry bulb outdoor air temperature	Campbell CS215	−40 °C to +70 °C	0,01 °C	°C
Outdoor Relative Humidity	Campbell CS215	0 to 100 %	0,03 %	%
Wind speed	Gill Instruments Windsonic	0 to 60 m/s	0.01 m/s	m/s
Wind direction in	Gill Instruments Windsonic	0–360°	0.1 °	°
Outdoor Air Pressure	Setra 278	600 to 1100 hPa	0.01 hPa	hPa
Global horizontal solar radiation	Kipp & Zonen CMP11	0 to 4000 W/m²	ISO 9060 Class A	W/m²

## Limitations

Our dataset has several limitations and biases to consider.

• During hot summer days one occupant consistently arrives early (around 7:30 AM local time) and opens all the windows.
• In general, all interior doors remain open all day long.
• Occupants sometimes change offices, meaning that between different years there are not the same occupants in each office throughout the test. Several sensors and metrics were not measured during instrumentation. The absence of CO_2_ sensors prevents us from determining occupancy, and there is a lack of sensors measuring air quality (e.g., VOCs) and airflow from natural ventilation.

No background survey has been conducted to gather information about the occupants, their preferences, or their habits.

There are no indoor air temperature measurements during the night, which would have been useful to assess the thermal impact of nighttime cooling. This lack of data is due to hardware limitations, as indoor temperature sensors only work when computers are switched on.

## Ethics Statement

The authors confirm that the ethical requirements for publishing data in Data in Brief have been read and understood and further confirm that the data collected does not involve human subjects, animal experiments, nor data collected from social media platforms.

## CRediT Author Statement

**Simon Bal-Fontaine:** Writing original draft, Writing Review and edit, Vizualization. **Pierre Bernaud:** Conceptualization, Investigation, Data curation, Writing Review and edit. **Kevin Campagna:** Writing original draft, Writing Review and edit, Vizualization, Data curation. **Hugo Coulandreau:** Conceptualization, Investigation, Data curation. **Aurélie Foucquier:** Conceptualization. **Arnaud Jay:** Conceptualization, Writing Review and edit. **Merveil Muanda Lutete:** Investigation, Data curation

## Data Availability

DataverseIn-situ Data of occupant behavior and thermal comfort in 11 offices in a naturally ventilated building during 6 summers (Original data).
